# Evaluation of Post-cholecystectomy Syndrome in Patients With Gallstone Disease: A Critical Appraisal

**DOI:** 10.7759/cureus.90965

**Published:** 2025-08-25

**Authors:** Khandakar R Rahman, Rida Fatima, Mareya Zeenat, Charlotte Williams, Azra Khatun, Otabek Yangibaev

**Affiliations:** 1 Department of General Surgery, Pilgrim Hospital, Boston, GBR

**Keywords:** conventional laparoscopic cholecystectomy, gallbladder diseases and gallstones, health education and awareness, post-cholecystectomy syndrome, post-operative complications

## Abstract

Background and aim: Laparoscopic cholecystectomy is the gold standard treatment for gallstones, offering a minimally invasive approach with quicker recovery times. Despite its effectiveness, a significant proportion of patients experience persistent symptoms post-surgery, collectively referred to as post-cholecystectomy syndrome (PCS). The incidence, causes, and management of PCS remain underexplored. This study aimed to evaluate the incidence of PCS among adult patients undergoing elective laparoscopic cholecystectomy for benign gallstone disease at Pilgrim Hospital, Boston, UK, and to identify potential contributing factors.

Methods: We conducted a retrospective study, reviewing data from patients who underwent elective laparoscopic cholecystectomy between January 1, 2024, and July 31, 2024. Data were collected through electronic records and patient questionnaires completed over the phone. The study analyzed patient demographics, symptoms post-surgery, and subsequent investigations and treatments.

Results: Out of 50 patients included in the study, 17 (34%) reported ongoing symptoms consistent with PCS. The most common symptoms were right upper quadrant (RUQ) pain (48%) and diarrhea (33%). Further investigations, including magnetic resonance cholangiopancreatography (MRCP) and esophagogastroduodenoscopy (OGD), were required for seven patients (41%) due to persistent symptoms. Five of the 17 patients (29%) were readmitted due to complications, such as infections and bile leaks. Despite these issues, 88% of PCS patients reported improved quality of life post-surgery.

Conclusion: Our study found that 34% of patients experienced persistent symptoms of PCS following laparoscopic cholecystectomy. While most reported an improvement in quality of life, a significant number required additional investigations and readmissions. The findings suggest that PCS remains an under-recognized condition and further research is needed to identify its underlying causes and improve diagnostic protocols. Standardized diagnostic criteria and long-term follow-up could help in better managing these patients.

## Introduction

Laparoscopic cholecystectomy has become the gold standard for treating gallstone disease due to its minimally invasive approach, reduced complication rates, and shorter recovery times [1]. Most patients expect complete symptom relief following this procedure; however, a significant proportion report persistent or new symptoms post-operatively [2]. When symptoms resembling the pre-operative state persist beyond surgery, this condition is termed post-cholecystectomy syndrome (PCS), with incidence rates ranging from 5% to 47% in the literature [3].

PCS encompasses a wide range of gastrointestinal complaints, including right upper quadrant (RUQ) pain, diarrhea, bloating, nausea, and vomiting [3,4]. Various etiologies have been proposed, such as retained or newly formed biliary stones, bile acid malabsorption, sphincter of Oddi dysfunction, and concurrent upper gastrointestinal disorders like gastroesophageal reflux disease (GORD) or peptic ulcers [5]. Despite advances in diagnostic imaging - including ultrasound (US), magnetic resonance cholangiopancreatography (MRCP), and endoscopic retrograde cholangiopancreatography (ERCP) - which demonstrate up to 98% accuracy in detecting choledocholithiasis, some underlying pathophysiological mechanisms of PCS may remain undetected pre-operatively [6].

Given the limitations of current diagnostic tools in predicting or preventing PCS, it is essential to investigate the patterns and risk factors contributing to symptom persistence following laparoscopic cholecystectomy. Moreover, understanding these outcomes is crucial for optimizing patient selection, improving pre-operative counseling, and refining post-operative care strategies.

This study primarily aimed to evaluate the incidence of PCS among adult patients undergoing elective laparoscopic cholecystectomy for benign gallstone disease at Pilgrim Hospital, Boston, UK, and to identify potential contributory factors. Additionally, we sought to assess the impact of laparoscopic cholecystectomy on post-operative outcomes, such as symptom resolution, quality of life, hospital readmissions and the need for further medical interventions in cases of uncomplicated gallstone disease.

## Materials and methods

This retrospective observational study was conducted in the Department of General Surgery at Pilgrim Hospital, Boston, UK, and included data from patients who underwent elective laparoscopic cholecystectomy between January 1, 2024, and July 31, 2024. The primary aim of the study was to find the incidence of post-cholecystectomy syndrome, defined as the persistence or emergence of gastrointestinal symptoms similar to those experienced pre-operatively, lasting beyond four weeks after surgery. Secondary aims included the identification of specific symptom patterns among PCS patients, evaluation of pre-operative and intra-operative factors that might influence symptom persistence, assessment of post-operative additional interventions (e.g., MRCP, OGD, or laparoscopy), post-operative readmissions, and subjective reports of changes in quality of life after surgery. The study was approved by the hospital audit board.

The total number of laparoscopic cholecystectomies performed during the study period was 150. Patients were selected for participation using simple random sampling. Microsoft Excel RAND function (Redmond, WA: Microsoft) was used to assign random numbers to all 150 eligible patients, and the first 50 patients who provided complete data were selected in ascending order of their assigned numbers. Any patient providing missing data were replaced by the next patient as per the previous random sampling. Patients were included if they were aged 18 years or older, had undergone an elective laparoscopic cholecystectomy for benign gallstone disease, and were available for post-operative follow-up (beyond four weeks of operation) through a structured telephone questionnaire (table in appendix). Patients were excluded if they had undergone emergency cholecystectomy, had gallbladder malignancy or other non-gallstone-related indications for surgery, had incomplete medical records, were lost to follow-up, or were unable to provide reliable information due to cognitive impairment or language barriers.

Clinical data were obtained through electronic medical records, which included information on pre-operative laboratory tests (e.g., liver function tests), imaging modalities (ultrasound, MRCP, ERCP), operative findings, post-operative complications, and readmissions. Additionally, the presence of ongoing gastrointestinal symptoms, such as right upper quadrant pain, diarrhea, nausea, bloating, acid reflux, etc., lasting more than four weeks, along with age, sex, clinical indication, pre-operative symptoms related to gallbladder disease, and symptom resolution post-surgery, were recorded using the questionnaire. The questionnaire recorded the number of gallbladder attacks before surgery, hospital or emergency admissions (excluding same-day emergency care), comorbidities, and pre-operative liver function tests were also documented. Information regarding pre-operative ERCP and presence of common bile duct stones, intra-operative complications, duration of surgery, post-operative length of stay, and readmissions within 30 days with reasons were recorded as well. Post-operative interventions, patient satisfaction, quality of life compared to before surgery, and patient response to follow-up were also captured. Verbal consent was obtained from all participants prior to the telephonic interview. All calls were conducted by trained resident doctors (FY1/FY2 level) under senior supervision. Telephone responses were documented during the call and verified by a second reviewer within 24 h. All responses were anonymized, and confidentiality was maintained throughout the study in accordance with institutional ethics guidelines.

All data were compiled and analyzed using Microsoft Excel and SPSS version 27.0 (Armonk, NY: IBM Corp.). Descriptive statistics were used to summarize patient demographics, clinical features, and symptom prevalence. Categorical variables were presented as frequencies and percentages, while continuous variables were reported as means with standard deviations. Comparative analyses between patients with and without PCS symptoms were conducted using chi-square or Fisher’s exact test for categorical variables. Normality of continuous variables (age and duration of surgery) was assessed using the Shapiro-Wilk test. For age, the test yielded a p-value of 0.6866 (W=0.9656), indicating that the data were approximately normally distributed. While raw values for duration of surgery were not available for formal testing, the mean and standard deviation (61.5±13.4 min for non-PCS, 58.2±15.0 min for PCS) suggested a roughly symmetric distribution without outliers. Additionally, the sample sizes in both groups were sufficient to rely on the central limit theorem. Therefore, the independent samples t-test was considered appropriate for comparing both age and surgical duration between PCS and non-PCS groups. A p-value of less than 0.05 was considered statistically significant.

## Results

Out of over 150 laparoscopic cholecystectomies performed during the study period, 50 patients were randomly selected for data collection (n=50). Among these, 17 patients (34%) reported persistent symptoms suggestive of post-cholecystectomy syndrome (PCS), although none had been officially diagnosed.

Table 1 presents the distribution of ongoing symptoms in these 17 patients, with some patients presenting with multiple symptoms. The most common symptom was right upper quadrant (RUQ) pain, reported by 10 patients (48%), followed by diarrhea in seven patients (33%), and other gastrointestinal symptoms, including nausea, bloating, and acidity, in four patients (19%).

**Table 1:** Number and percentage of patients (n=50) with ongoing signs and symptoms post-surgery. RUQ: right upper quadrant

Symptoms	Number	Percentage
RUQ pain	10	48%
Diarrhea	7	33%
Other (nausea/vomiting, etc.)	4	19%

The indications for laparoscopic cholecystectomy are detailed in Table 2. The most common indication was symptomatic gallstones or biliary colic (59%), followed by acute calculous cholecystitis (29%), and gallstone pancreatitis (12%).

**Table 2:** Indications for laparoscopic cholecystectomy among study participants (n=50).

Indications	Number	Percentage
Symptomatic gallstones	10	59%
Acute calculous cholecystitis	5	29%
Gallstone pancreatitis	2	12%

Table 3 compares clinical and surgical parameters between patients with ongoing symptoms (n=17) and those without (n=33). While most differences did not reach statistical significance, two parameters - anxiety/fibromyalgia and readmission rates - were significantly associated with persistent symptoms. Anxiety or fibromyalgia was present in 18% of symptomatic patients but was not reported in any asymptomatic patients (p=0.035). Similarly, readmission was more frequent in the symptomatic group (24% vs. 3%, p=0.040). While descriptive differences are noted in variables, such as pre-operative ERCP use (less common in symptomatic patients: 12% vs. 30%) did not reach statistical significance. However, these trends may still be clinically relevant and warrant further exploration in a larger cohort. Regarding continuous variables, age and duration of surgery were compared using independent samples t-tests. As shown in Table 4, no significant differences were observed between groups in either parameter.

**Table 3:** Comparison of clinical and surgical parameters in patients with and without PCS (n=50). *Statistically significant at p<0.05. Comparative analyses were conducted using the chi-square test or Fisher’s exact test as appropriate. PCS: post-cholecystectomy syndrome; LFTs: liver function tests; ERCP: endoscopic retrograde cholangiopancreatography

Parameters	Without PCS (n=33), n (%)	With PCS (n=17), n (%)	p-Value	Test statistic
Obesity	9 (27%)	5 (29%)	1.000	Fisher’s exact
GORD/hernia	6 (18%)	3 (18%)	1.000	Fisher’s exact
Anxiety/fibromyalgia	0 (0%)	3 (18%)	0.035*	Fisher’s exact
Day case surgery	26 (78%)	14 (82%)	1.000	Fisher’s exact
Pre-operative ERCP	10 (30%)	2 (12%)	0.181	χ²=1.79
Pre-operative deranged LFTs	12 (36%)	5 (29%)	0.757	χ²=0.10
Readmission	1 (3%)	4 (24%)	0.040*	Fisher’s exact

**Table 4:** Independent samples t-test results comparing continuous clinical variables between PCS and non-PCS patients (n=50). PCS: post-cholecystectomy syndrome

Continuous Variables	Without PCS (mean±SD)	With PCS (mean±SD)	p-Value	t-Value
Age (years)	42.8±11.6	45.3±12.1	0.421	0.81
Duration of surgery (min)	61.5±13.4	58.2±15.0	0.476	-0.72

## Discussion

This study offers valuable insights into the outcomes of laparoscopic cholecystectomy (LC), particularly focusing on the persistence of post-cholecystectomy syndrome (PCS) symptoms. Although LC remains the gold standard for treating gallstone disease due to its minimal invasiveness and faster recovery times [1], our findings show that 34% of patients (17 out of 50) continued to experience PCS symptoms post-operatively, consistent with previously reported ranges of 5-47% [2,3]. The statistically significantly higher number of readmissions for patients with ongoing PCS further reinforces the notion that a significant number of patients may not experience full symptom resolution after surgery.

The most prevalent ongoing symptom was right upper quadrant (RUQ) pain (48%), followed by diarrhea (33%) and other gastrointestinal symptoms, such as bloating, acidity, and nausea (19%), as detailed in Table 1. These results reflect findings from prior studies that emphasized the multifactorial and sometimes elusive etiology of PCS symptoms, which include mechanical, functional, and psychosomatic contributors [2,5].

Several pathophysiological mechanisms have been proposed to explain PCS. One common theory involves bile reflux into the stomach and esophagus following removal of the gallbladder, potentially causing gastritis, duodenitis, or exacerbation of gastroesophageal reflux disease (GORD) [5]. Other mechanisms include changes in gastrointestinal motility, visceral hypersensitivity, and altered enterohepatic circulation of bile acids [3,5]. These factors may individually or collectively contribute to persistent symptoms, especially when no overt structural abnormalities are identified on imaging.

A notable but often underrecognized contributor to PCS is sphincter of Oddi dysfunction (SOD), a functional biliary motility disorder. SOD is characterized by abnormal motility or hypertensive contractions of the sphincter, leading to intermittent biliary obstruction and recurrent pain episodes [6]. This is particularly relevant in patients whose imaging (e.g., MRCP or ERCP) fails to reveal residual stones or strictures but who continue to report biliary-type pain.

Regarding pre-operative investigations, our study found that patients who underwent pre-operative ERCP were less likely to develop PCS (12% vs. 30%), although this difference did not reach statistical significance. A possible explanation for this observation lies in the anatomical configuration of the biliary and pancreatic ducts. It is known that the common bile duct (CBD) and the main pancreatic duct (MPD) converge at the ampulla of Vater in approximately 60% of individuals, forming a standard single-channel anatomy. In about 38% of cases, they form a "double-barrel" structure at the duodenal papilla, while true separate openings occur in only around 2% of the population [7]. This anatomical arrangement may play a significant role in the pathogenesis of gallstone disease (GSD) and contribute to persistent post-operative symptoms. The lower incidence of PCS among patients with a history of ERCP in our cohort supports this hypothesis, suggesting that ERCP - particularly when it involves papillosphincterotomy (PST) - has a potentially protective effect regarding PCS development by relieving biliary pressure [8,9]. Notably, deranged liver function tests (LFTs) were not predictive of ongoing symptoms, further underscoring the limitations of current diagnostic algorithms in identifying patients at risk for PCS.

Notably, 18% of symptomatic patients in our cohort had comorbid anxiety or fibromyalgia, whereas none of the asymptomatic group had these conditions. This statistically significant observation aligns with the growing body of literature linking functional gastrointestinal disorders with disorders of gut-brain interaction, often mediated by psychological stressors [10,11]. According to the Rome IV criteria, these psychosomatic links may result in symptom amplification, even in the absence of clear anatomic pathology [10]. Therefore, psychological screening and holistic patient evaluation might be beneficial components of peri-operative care.

The findings from this study therefore indicate that although modalities such as MRCP, ERCP, and other pre-operative investigations boast high accuracy for detecting gallstone diseases, including choledocholithiasis (up to 98% in some studies), their predictive value for PCS is limited, especially when the syndrome stems from functional or non-biliary causes [12,13]. This may suggest that despite the high accuracy of these comprehensive investigation modalities, their diagnostic ability can be somewhat limited, indicating that more accurate diagnostic technologies are required to be developed, while also suggesting that current diagnostic approaches require revision and advancement.

Limitations of this study include its retrospective nature, small sample size (n=50) in a single centre, and reliance on patient-reported outcomes, predisposing to recall bias, without formal diagnostic criteria for PCS. Structured follow-ups for a uniform post-operative duration for all participants would probably provide more reliable and detailed data as well. Furthermore, no validated questionnaire or scoring system was employed to grade symptom severity, nor was any multivariable analysis done on the patient cohort. Ensuring that these are incorporated in future studies could improve comparability. Standardized QoL assessment tools, such as the Gastrointestinal Quality of Life Index (GIQLI), are helpful in objectively measuring post-operative outcomes and may guide questionnaire build-up, follow-up strategies, and supportive interventions.

While laparoscopic cholecystectomy is associated with high overall satisfaction rates, up to 20-30% of patients report persistent dyspepsia or vague abdominal discomfort, which can impact their perceived health status [14,15]. Despite its limitations, our study underscores the need for greater recognition of PCS and suggests avenues for improving surgical outcomes. Identifying at-risk individuals - especially those with functional disorders, comorbid psychiatric conditions, or atypical symptom profiles - and adequate pre-operative investigations, including a low threshold for pre-operative ERCP, should be considered. Future studies with larger cohorts and longer follow-up durations are essential to validate these findings and develop evidence-based protocols for the prevention and management of PCS.

## Conclusions

This study found that 34% of patients who underwent laparoscopic cholecystectomy developed persistent PCS symptoms. While 10% required readmission, none were directly due to PCS, although 80% of readmitted patients were from the PCS group. It highlights the fact that, although laparoscopic cholecystectomy remains an effective treatment for gallstone disease, significantly improving symptoms and quality of life for most patients, a notable proportion - approximately one-third - experience persistent symptoms, such as right upper quadrant pain and diarrhea, which are hallmarks of post-cholecystectomy syndrome. While no statistically significant predictors were identified, trends suggest that the presence of psychosomatic conditions may contribute to symptom persistence, while pre-operative ERCP might have a protective influence. These findings highlight the importance of thorough pre-operative evaluation, patient counseling, and tailored post-operative follow-up to better manage and potentially reduce the incidence of PCS. Further large-scale prospective studies are warranted to identify definitive risk factors and optimize management strategies.

## Appendices

**Table 5:** Post-cholecystectomy audit questionnaire. ERCP: endoscopic retrograde cholangiopancreatography

Sections	Questions	Responses
1. Patient demographics	Age	________
	Gender	________
	BMI	________
	Known allergies	________
2. Clinical background and pre-operative factors	Primary indication for cholecystectomy	☐ Symptomatic gallstones / ☐ Biliary colic / ☐ Acute cholecystitis / ☐ Other: ________
	History of digestive/biliary issues pre-surgery	☐ Yes / ☐ No
	If yes, specify	________
	Pre-operative symptoms related to gallbladder disease	☐ Abdominal pain / ☐ Nausea/vomiting / ☐ Dyspepsia/bloating / ☐ Diarrhea / ☐ Bloating / ☐ Jaundice / ☐ Other: ________
	Did pre-operative symptoms resolve post-surgery?	☐ Yes / ☐ No (If no, specify persistent symptoms) ________
	Number of gallbladder attacks before surgery	With hospital admission: ________ / Without hospital admission: ________
	Length of hospital stay pre-surgery (for acute attacks)	________ days
	Comorbidities	☐ Diabetes mellitus / ☐ Hypertension / ☐ Cardiovascular disease / ☐ Chronic kidney disease / ☐ Hematological conditions / ☐ Other: ________
	Pre-operative investigations findings	CBD stones present: ☐ Yes / ☐ No | Deranged LFTs: ☐ Yes / ☐ No
	ERCP performed pre-operatively?	☐ Yes / ☐ No
3. Surgery details (peri-operative factors)	Type of cholecystectomy performed	Setting: ☐ Elective / ☐ Emergency | Method: ☐ Laparoscopic / ☐ Open / ☐ Converted to open
	Intra-operative complications	Gallbladder perforation: ☐ Yes / ☐ No | Bile leak: ☐ Yes / ☐ No | Other: ________
	Duration of surgery	________ minutes
	Length of hospital stay post-operation	________ days
4. Post-operative outcomes	Readmission within 30 days post-cholecystectomy	☐ Yes / ☐ No
	If yes, reason for readmission	☐ Pain / ☐ Wound infection / ☐ Bile leak / ☐ Intra-abdominal collection / ☐ Other: ________
	Development of post-cholecystectomy syndrome (PCS) symptoms	☐ Abdominal pain / ☐ Diarrhea / ☐ Dyspepsia / ☐ Bloating / ☐ Other: ________
	Was PCS officially diagnosed?	☐ Yes / ☐ No
	If yes, symptoms and treatment approach	________
5. Emergency admissions and timelines of surgery	Emergency admission for gallstone-associated pancreatitis?	☐ Yes / ☐ No
	If yes, was a cholecystectomy performed within 14 days of discharge?	☐ Yes / ☐ No
	If no, reason for delay	☐ Lack of resources / ☐ Other: ________
6. Follow-up and PCS development	Resolution of symptoms at follow-up	☐ Complete resolution / ☐ Persistent symptoms (specify) ________
	Total number of follow-up visits post-surgery	________
	Additional interventions or treatments post-surgery for PCS	☐ Medications / ☐ Dietary changes / ☐ Further operations/procedures / ☐ Other: ________
7. Patient satisfaction and quality of life post-surgery (optional section)	Patient satisfaction with the surgery outcome	☐ 1. Very dissatisfied / ☐ 2. Dissatisfied / ☐ 3. Neutral / ☐ 4. Satisfied / ☐ 5. Very satisfied
	Patient comments on quality of life post-surgery	________
